# SET/MYND Lysine Methyltransferases Regulate Gene Transcription and Protein Activity

**DOI:** 10.3390/genes2010210

**Published:** 2011-02-21

**Authors:** Kristin Leinhart, Mark Brown

**Affiliations:** Colorado State University, Fort Collins, CO 80523, USA; E-Mail: Kjleinha@rams.colostate.edu

**Keywords:** SET, MYND, Smyd1, Smyd2, Smyd3, transcriptional regulation, chromatin modifications, epigenetics, tumorigenesis

## Abstract

The SET and MYND (SMYD) family of lysine methyltransferases is defined by a SET domain that is split into two segments by a MYND domain, followed by a cysteine-rich post-SET domain. While members of the SMYD family are important in the SET-mediated regulation of gene transcription, pathological consequences have also been associated with aberrant expression of SMYD proteins. The last decade has witnessed a rapid increase in the studies and corresponding understanding of these highly impactful enzymes. Herein, we review the current body of knowledge related to the SMYD family of lysine methyltransferases and their role in transcriptional regulation, epigenetics, and tumorigenesis.

## 1. Introduction

From regulated gene expression to mitosis, chromatin acts as a structurally flexible repository of the genome [1]. In this manifestation, an entire chromosome is sequentially compacted through a series of highly ordered packaging while distinct regions of DNA are selectively made accessible to transcriptional complexes [2,3]. Thus, chromatin maintains a dynamic architecture that allows approximately 2 m of DNA to be condensed in the nucleus while retaining a remarkable degree of functionality [4,5]. At its foundation, chromatin consists of a succession of nucleosomes, the basic structural units [6], consisting of 146 base pairs of DNA, wrapped 1.7 times around an octamer of core histones and separated by a linker region of approximately 50 base pairs. The primary histones involved in the assembly of a nucleosome are histones H2A, H2B, H3 and H4. Histone tails interact with the poly-anionic backbone of the core DNA, marginally contributing to nucleosomal stability [7]. Therefore, regulation of chromatin structure and transcription is often mediated through post-translational modifications that alter specific residues along these tails [8]. These modifications can affect the accessibility of nuclear factors to DNA or induce the recruitment of such factors to transcriptional or chromatin assembly pathways [9,10].

Histone tail alterations encompass the greatest range of variation in epigenetic regulation, encompassing more than 50 known sites of modification [11,12]. Histones are subject to several forms of post-translational modification, including methylation, citrullination, acetylation, phosphorylation, SUMOylation and ADP-ribosylation [13]. These modifications impart biological consequences by acting as marks for the specific recruitment of regulatory complexes and affecting the structure of the nucleosome. Acting in concert, the combination of different histone modifications is thought to constitute a “histone code” that is interpreted in the form of specific nuclear events [14,15]. Although the interplay among various histone modifications is still largely nebulous, a paradigm is rapidly emerging whereby methylation, acetylation, or phosphorylation at independent sites work in tandem with other such modifications to convey unique biological consequences [16]. Such crosstalk has already been clearly demonstrated by a number of findings including the cooperation between acetylation and phosphorylation of histone H3 during the cell cycle [17], the correlation between acetylation and arginine methylation in the regulation of estrogen-responsive genes [18], and the competition between methylation and acetylation of histone H3, lysine 9 toward the establishment or disruption of heterochromatin [19]. As new studies continue to highlight the importance of crosstalk in chromatin signaling, our early understanding of singular histone modifications have yielded to a more delicate model in which minor variations in broad patterns of modifications impart distinct outcomes.

While acetylation of histone tails is largely ephemeral in nature, histone methylation is widely observed to be a mark that confers long-standing epigenetic memory [20]. Mounting evidence suggests that histone lysine methylation is a critical factor in such pathways as transcriptional regulation, X chromosome inactivation, DNA methylation, and the formation of heterochromatin [21–23]. Catalyzed by histone methyltransferases, this modification ultimately mediates either gene activation or silencing, in a residue-dependent manner [20]. The level of specificity is heightened by the variation in biological consequences associated with whether a residue is mono-, di-, or tri-methylated [24,25]. It has also been reported that many transient histone modifications work in tandem with histone lysine methylation, further increasing the potential complexity of this epigenetic modification [1].

Most histone lysine methyltransferases catalyze methyl transfer by way of the SET domain, a module encoded within many proteins that regulate diverse processes, including some critical for development and proper progression of the cell cycle [14,23,26]. Residue-specific histone lysine methylation typically correlates with distinct states of gene expression [27]. Most of the known targeted lysines of histone methyltransferases occur on histone H3 which thereby serves as a conduit of epigenetic regulation. In general, lysine methylation at histone H3, lysine 9 (H3K9), H3K27, or H4K20 corresponds with gene silencing, whereas methylation of H3K4, H3K36, or H3K79 is associated with actively transcribed genes. However, these paradigms are far too narrow to encompass the growing intricacies of the histone code [27]. Recent evidence implicates histone methylation in the recruitment of chromatin remodeling complexes, as is the case with CHD1, an ATP-dependent chromatin remodeling factor that specifically binds methylated H3K4 [28]. Once thought to be a permanent modification, enzymes have been identified that are capable of reversing histone methylation at specific sites [20,29].

## 2. SMYD Family

**Figure 1:**
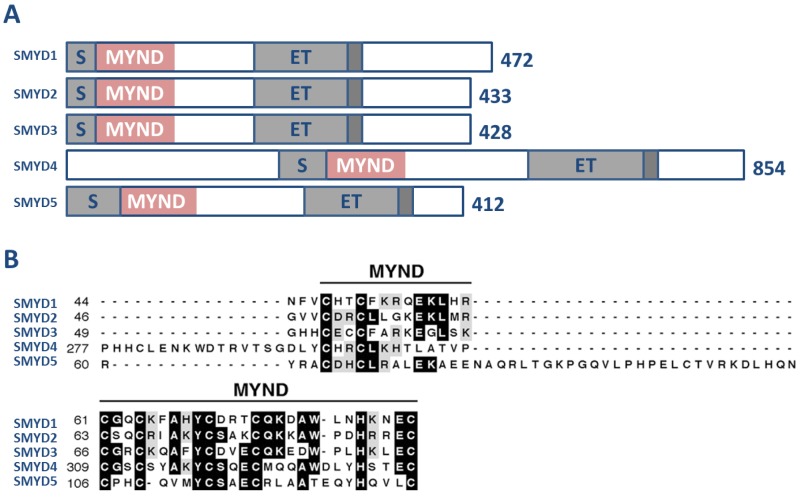
(**A**) Schematic representation of the five mammalian SMYD proteins. The split SET domain is shown in light gray; the MYND domain is shown in pink; the cysteine-rich post-SET domain is shown in dark gray; (**B**) ClustalW and BOXSHADE programs were used for alignment and shading of the MYND domains associated with each of the five SMYD proteins.

The SMYD family comprises a subset of five SET domain-containing proteins that have unique domain architecture. Specifically, this family of proteins is defined by a SET domain that is split into two segments by a MYND domain, followed by a cysteine-rich post-SET domain (Figure 1A) [30,31]. The SET domain is responsible for the methylation of lysine residues on target proteins [32–37] and, indeed, the SET domains of SMYDs 1, 2, and 3 have been confirmed to be active catalytic domains despite the split nature of their architecture [14,23,31,38,39].

The MYND domain of SMYD proteins (Figure 1B) encompasses a putative zinc-finger motif that facilitates protein-protein interactions. This domain is present in several other transcriptional regulators where it is known to contribute in developmental processes [40,41]. Interface at the MYND domain occurs through a PXLXP motif in the associating protein. For example, the associations of SMYD1 with HDACs and the transcription factor, skNAC, are mediated through these sites [30,42]. The MYND domain is the key feature distinguishing SMYDs from all other SET domain-containing proteins.

Thorough characterizations have not yet been conducted for SMYDs 4 or 5. Thus the catalytic activity has not been determined for those proteins. In fact, other than their identification [31] and data from Expressed Sequence Tags suggesting that they are expressed in a wide range of normal, tumor, and diseased tissues, little is known about SMYDs 4 or 5. The expression of SMYD5 appears to be responsive to retinoic acid which could have broad implications regarding the regulation of the SMYD family [43]. Computational analyses based on BLAST comparisons of the SMYD family using the protein database of the National Center for Biotechnology Information revealed that while the SET and MYND domains of the SMYD family members contain a high level of identity and similarity, SMYDs 4 and 5 are much less conserved in their other domains [31]. The high degree of conservation in their SET and MYND domains suggests that these are likely lysine methyltransferases with the capacity to bind proteins containing the MYND cognate motif, PXLXP. As they are all but absent from the literature, this review will not cover SMYDs 4 or 5.

## 3. SMYD1-Regulator of Heart and Skeletal Muscle Development

Gottlieb *et al.* first identified SMYD1 as a cardiac- and skeletal muscle-specific protein that is critical for cell differentiation and heart morphogenesis during embryonic development [30]. Targeted deletion of SMYD1 was shown to hinder the differentiation of cardiomyocytes leading to malformation of the right ventricle. Using reporter assays, they demonstrated that SMYD1 functioned as an HDAC-dependent transcriptional repressor. The same group later demonstrated that SMYD1 interacts with the muscle-specific transcription factor, skNAC, by way of a MYND-PXLXP interaction between SMYD1 and skNAC, respectively [42]. SMYD1 has more recently been shown to be an immediate target of the transcription factor, MEF2C during cardiac morphogenesis [44].

In 2006, Tan *et al.* published the first confirmation that the SET domain of SMYD1 is a catalytically active lysine methyltransferase [39]. Specifically, they determined that SMYD1 targets histone 3, lysine 4. In accord with previous findings associating SMYD1 with developmental processes of cardiac and skeletal muscle, this group further demonstrated that SMYD1 is essential for muscle contraction and myofibril organization.

This year, Sirinupong *et al.* published the crystal structure for SMYD1 [45] providing the first detailed structural analysis for a SET/MYND domain containing protein. Their analysis illustrates the mechanism by which this family is capable of methylating target lysines despite the split nature of their SET domains. They also provide a plausible conjecture suggesting that the intervening sequence and unique C-terminal domain (CTD) contribute to an autoinhibitory mechanism. That domain is shown to facilitate the adoption of two distinct conformations thereby regulating the catalytic function of SMYD1. Given the partial occupation of the CTD in a site typically occupied by the pre-SET domain of other SET proteins, it is conjectured that the CTD may contribute to the stabilization of the SET domain in SMYD1. Finally, the resolution of the MYND domain confirms the structural basis for the role of that domain in guiding the protein-protein interactions of SMYD1. Lacking in their analysis is a thorough comparison of the SMYD1 SET with the SET domain of other proteins. Indeed, crystal analyses have been published for a number of other SET proteins [32–37]. Given the unique architecture of the SMYD family SET domains, an attempt to align and overlay the SET of SMYD1 with other published SETs could provide a great deal of insight into the structural and mechanistic properties associated with the methyltransferase activity of SET domains.

## 4. SMYD2—Lysine Methyltransferase and Regulator of Tumor Suppressors

The identification and characterization of SMYD2 was published by Brown *et al.* in 2006 [31]. In that report, SMYD2 was identified as a histone 3, lysine 36-specific methyltransferase. In contrast to SMYD1, SMYD2 was observed to be broadly expressed across several tissues with highest expression in the heart and the hypothalamus. Although most evidence suggests that histone 3, lysine 36 methylation is associated with actively transcribed genes, SMYD2 was shown to repress transcription in reporter assays. The Sin3A histone deacetylase complex, which has been linked to histone 3, lysine 36 methylation within the coding regions of active genes [46,47] was shown to interact with SMYD2. This HDAC interaction provides a likely explanation for the observation of transcriptional repression in *in vitro* reporter assays where the effect of chromatin modifications imparted by SMYD2 on the recruitment of other factors could not be observed. Further evidence linking SMYD2 to transcriptional regulation was reported in the observation that SMYD2 interacts with RNA polymerase II and the RNA helicase, HELZ [48].

SMYD2 has also been shown to catalyze methylation of non-histone targets. Control of spatial and temporal expression of SMYD2 has been proven critical, as methylation of p53 at lysine 370 by SMYD2 was observed to repress the activity of p53 [49]. Thus, SMYD2 has the capacity to function as an oncogene by eliminating the tumor suppressor functionality of p53. SMYD2 has more recently been linked to the regulation of the retinoblastoma tumor suppressor (RB) through its methylation of RB at lysine 860 [50]. This modification has been shown to facilitate interaction with the transcriptional repressor, L3MBTL1 via the methyl binding domain of that protein. There is much left to uncover regarding the role of SMYD2 in oncogenesis. Its role in the regulation of two such critical pathways in tumor suppression make it a promising target for therapeutics.

## 5. SMYD3—Transcriptional Regulation and Tumorigenesis

SMYD3 was the first member of the SMYD family for which the catalytic activity of the SET domain was confirmed. SMYD3 methylates histone 3, lysine 4 and has a role in the regulation of transcription through its association with an RNA polymerase complex [38]. SMYD3 is overexpressed in most hepatocellular (HCC) and colorectal carcinomas (CRC) [38] and its upregulation has been proven to be critical in the proliferation of breast cancer cells [51]. Through microarray analyses, 80 genes have been identified that display altered gene expression in the presence of Smyd3 [38]. Notably, one of these is Nkx2.8, a homeobox gene that exhibits upregulation in hepatocellular malignancies [52]. Other affected genes include cell cycle regulators, oncogenes, and several that are critical in developmental processes [38]. In concert with the idea that SMYD3 is a transcriptional activator, it forms a complex with RNA polymerase II through its interaction with the RNA helicase, HELZ, and it was also shown to bind DNA directly by way of a sequence found in the promoter of Nkx2.8 [38]. These findings not only provide targets for the histone 3, lysine 4 enzymatic activity of SMYD3 but they also implicate two methods for its direct interaction with those genes [53].

The findings that Smyd3 is substantially upregulated in most CRCs [54], HCCs [55], and breast cancer tissues [51] support a paradigm in which aberrant expression of chromatin-modifying enzymes, leading to a disturbance in established epigenetic patterns, can ultimately result in tumorigenesis. The recognition of the role of SMYD3 in tumorigenesis has led to studies regarding the effects of the knockdown of SMYD3 in cancer cells. RNA Interference in many types of tumor cells, leading to knockdown of SMYD3, has been observed to inhibit cell proliferation [38,51]. Thus, SMYD3 has emerged as yet another promising target for therapeutic intervention in cancer.

## 6. Conclusions

The SMYD Family is a group of SET and MYND domain-containing transcriptional regulators that function, at least partly, through histone modifications. Future research on SMYD proteins, with strong emphasis on the unique organismal context, will shed light onto the biological functions of SMYD family proteins. Such emphasis may reveal new insights into the relationships between protein modifications and the development and differentiation of tissues and organisms as well as pathways through which aberrant activity of protein modifiers lead to tumorigenesis.
